# American Public Health Association

**Published:** 1879-12

**Authors:** 


### Editorial.

### AMERICAN PUBLIC HEALTH ASSOCIATION.

This very useful and philanthropic body met in full force at Nashville recently, and held a harmonious meeting. Useful, yes useful, in the indorsement and support of another National body, whose ostensible object is the protection of American citizens against foreign diseases, the chief of which is said to be yellow fever. A big thing has been inaugurated by the Surgeon General of Marine Hospital Service, and the A. P. H. Association is necessary to insure the requisite amount of public and political influence for the successful operation of the National Board of Health, which was created at the suggestion of the said Surgeon General, to receive and handle the money necessary to carry out his great quarantine project. Both bodies then, are the creatures of this floating coast and river service, the head of which conceived the grand scheme of a central quarantine power, ostensibly to protect our people against disease, but which really operates injuriously to Southern commerce and travel, without compensating benefit. That quarantine, for protection against yellow fever, is a total failure, the whole world has abundant evidence in the scourge at Memphis the past summer. The people began to look upon and talk of the National Board of Health as a failure in the accomplishment of the object for which it was supposed to have been created. Even newspapers that had been bold and loud in praise of the great National benificence, frankly acknowledged the failure. At this critical period in the existence of the quarantine bonanza, the approaching meeting of the health association, was doubtless looked to as the auspicious time for smothering the sparks of common sense about to be fanned into a flame, by positive evidence of local origin.

We should not, therefore, wonder that the meeting was large, and made up of quarantine material; that a majority was secured who would think, talk and sing of quarantine, so loud and long that those opposed would not dare raise their voice against it.

Moreover, membership in these great National bodies, with over half a million of dollars at their disposal, is sought by those fond of notoriety and a name in the National Archives. Hence, blatant advocates for quarantine were found at the meeting, who would not otherwise have been present. Besides, many paying offices are filled those who feel an interest in promoting the universal acceptance of the quarantine system, as inaugurated and being carried out by the head of Marine Hospital Service, through the National board of health, supported and encouraged by the American Public Health Association.

We thus speak of these associations and influences, because they all work to the same object. The establishment of a central quarantine power to control commerce and travel, is first and last, with all. Discussion of the utility and propriety of quarantine is, to a large extent, ignored. The associations will not hear nor talk of anything but quarantine. If investigation as to the origin of fever be proposed, the discussion is made to turn at once upon the best mode of quarantine.

